# Functional alignment with robotic-assisted total knee arthroplasty is associated with reduced early postoperative pain and alignment outliers compared to mechanical alignment with conventional total knee arthroplasty: a prospective cohort study

**DOI:** 10.1051/sicotj/2026058

**Published:** 2026-09-18

**Authors:** Wai Kit Wong, Naim Che-Kamaruddin, Patrick Ze En Ng, Shi Yao Chong, Siang Leon Chin, Hwa Sen Chua

**Affiliations:** 1 Orthopaedic Centre of Excellence, Sunway Medical Centre, Bandar Sunway 47500 Subang Jaya Selangor Malaysia; 2 Clinical Research Centre, Sunway Medical Centre, Bandar Sunway 47500 Subang Jaya Selangor Malaysia; 3 IMU University 126, Jalan Jalil Perkasa 19, Bukit Jalil 57000 Kuala Lumpur Malaysia; 4 Sunway Medical Centre Bandar Sunway 47500 Subang Jaya Selangor Malaysia; 5 Department of Orthopaedics, Hospital Sri Aman, Jalan Mangga, Pekan Sri Aman 95000 Simanggang Sarawak Malaysia

**Keywords:** Robotic-assisted total knee arthroplasty (RATKA), MAKO, Patient-reported outcome measures, Outliers, Rotational alignment

## Abstract

*Introduction*: Robotic-assisted total knee arthroplasty (RATKA) has been introduced to enhance surgical precision, alignment, and soft-tissue balancing, thus enabling the advent of numerous alignment philosophies including functional alignment (FA). This study aimed to compare early clinical outcomes, patient-reported outcome measures (PROMs), and radiographic accuracy brought about by RATKA using FA principles against the current reference standard of conventional total knee arthroplasty (TKA) and mechanical alignment (MA). *Methods*: This prospective, non-randomized cohort study included 45 patients undergoing primary unilateral TKA (30 RATKA, 15 conventional). Outcomes assessed were Visual Analogue Scale (VAS), Knee Society Score (KSS), and Knee injury and Osteoarthritis Outcome Score (KOOS) preoperatively and up to 1 year postoperatively. Forgotten Joint Score-12 (FJS-12) was evaluated at 1 year. Radiographic parameters included hip-knee-ankle angle (HKA), medial proximal tibial angle (MPTA), lateral distal femoral angle (LDFA), and femoral component rotation. Alignment outliers were defined as a deviation of > ±3° from the target. Statistical significance was set at *p* < 0.05. *Results*: RATKA resulted in significantly lower VAS pain scores at 2 weeks and 3 months (*p* < 0.05), with a consistent trend toward reduced pain at other timepoints. PROMs demonstrated improvements favoring RATKA across several KSS and KOOS subdomains at selected intervals. FJS-12 was higher in the RATKA group but did not reach statistical significance (*p* = 0.12). Radiographic alignment was comparable between groups except for femoral component rotation, which showed greater external rotation in the conventional cohort (*p* = 0.009). RATKA significantly reduced alignment outliers (10.0% vs 40.0%, *p* = 0.042). No complications were observed. *Conclusion*: The adoption of RATKA and functional alignment is associated with reduced early postoperative pain, modest improvements in PROMs, and improved consistency in achieving target alignment, without increased complications.

## Introduction

Total knee arthroplasty (TKA) adopting mechanical alignment principles remains the gold standard treatment for end-stage knee osteoarthritis (OA), with registry data demonstrating excellent implant survivorship [1]. Despite this, patient satisfaction rates remain lower than those observed following total hip arthroplasty, with contemporary studies reporting satisfaction rates of approximately 90% [2, 3].

The growth of robotic-assisted total knee arthroplasty (RATKA) has coincided with growing recognition of constitutional alignment and dynamic soft-tissue balancing [4–6]. Robotic systems enable detailed preoperative planning and real-time intra-operative assessment, facilitating the development and implementation of individualized alignment strategies that are not achievable with conventional techniques [7, 8].

These advancements aim to improve clinical outcomes and patient satisfaction following TKA. The primary aim of this prospective study was to evaluate early postoperative patient-reported outcome measures (PROMs) following RATKA. Secondary objectives included assessment of the accuracy of implant positioning and alignment outlier rates compared to conventional TKA. We hypothesized that RATKA would result in reduced postoperative pain, improved PROMs, more accurate implant positioning, and fewer alignment outliers.

## Materials and methods

### Patients

This is a prospective non-randomized cohort study conducted after obtaining ethical clearance from the Sunway Medical Centre Independent Research Ethics Committee (SREC No.: 018/2023/IND/ER) and was conducted in accordance with the principles of the Declaration of Helsinki. Inclusion criteria included patients older than 18 years old, suffering from end-stage bi- or tri-compartmental knee osteoarthritis (OA) who have exhausted conservative management and would be undertaking a unilateral primary TKA. Exclusion criteria include revision surgery, conversion from a previous unicompartmental knee arthroplasty (UKA) to TKA, simultaneous bilateral primary TKA, significant bone loss requiring additional implants/augments or those with implants of increased constraint, recent arthrotomy or intra-articular injection within the preceding 6 months, history of infection of the same knee, and non-compliance with follow-up or post-operative rehabilitation programs. We offered study enrollment to all eligible patients who fulfilled the inclusion and exclusion criteria as listed above.

We initially aimed for a 1:1 recruitment ratio between study arms. However, due to patient preference in a private healthcare setting, a substantially higher proportion elected to undergo robotic-assisted total knee arthroplasty (RATKA), and recruitment was adapted to an approximate 2:1 ratio. The patients in the robotic arm underwent RATKA using the Robotic Arm Interactive Orthopedic System (RIO; MAKO Stryker, Fort Lauderdale, Florida). A priori sample size was calculated following previously published studies evaluating functional outcomes following RATKA versus conventional TKA [9]. Assuming a moderate effect size (Cohen’s *d* = 0.8), a two-sided alpha level of 0.05, and 80% power, the achieved sample size (*n* = 45; 2:1 allocation) is adequate to detect moderate differences between groups, although smaller effects may be underpowered. Enrollment was therefore concluded upon achieving the target sample size for the conventional TKA arm, resulting in a total study population of 45 patients. All procedures were performed by a single high-volume arthroplasty surgeon experienced in both conventional and robotic techniques.

### Clinical assessment

Patients were assessed preoperatively and postoperatively at 2 weeks, 6 weeks, 3 months, and 1 year. Outcomes included Visual Analogue Scale (VAS), Knee Society Score (KSS, 2011), and Knee injury and Osteoarthritis Outcome Score (KOOS). Forgotten Joint Score-12 (FJS-12) was assessed at 1 year. All clinical assessments were performed by the senior author to minimize inter-observer variability. Patients completed the Patient-Reported Outcomes Measures themselves.

We additionally assessed pain using the VAS in the immediate peri-operative period prior to discharge. This was done by trained Orthopaedic nurses and recorded in the electronic medical records system.

### Radiological assessment

Pre-operative radiographs include an AP, lateral, and skyline views of the affected knee along with a long limb film. For patients in the RATKA arm, they required a pre-operative computed tomography (CT) scan as mandated by the Mako robotic system. The complete set of radiographs and CT scans was repeated at 6 weeks post-op for all patients in this study, including those in the conventional arm, who understood that the post-operative CT was purely for research purposes, specifically the assessment of rotational accuracy.

All images were hosted on a PACS system. Measurements were performed using the bundled measurement instruments. Parameters measured included the Hip-Knee-Ankle axis (HKA), lateral distal femoral angle (LDFA), medial proximal tibial angle (MPTA), and rotational alignment of the femoral component, which was referenced against the trans-epicondylar axis (TEA). Additionally, we calculated the arithmetic HKA (aHKA) and Joint Line Obliquity (JLO), which are derived using the following equations: aHKA = MPTA − LDFA and JLO = MPTA + LDFA [5]. For the RATKA cohort, these were then compared to intra-operative values obtained from the MAKO console. For the conventional arm, these values were compared to the expected values when aiming for a neutral HKA as per mechanical alignment principles. We also calculated the outlier rate between both cohorts, taking target alignment ±3° as normal limits.

### Data analysis

Continuous variables are presented as mean ± standard deviation (SD) for normally distributed data, and median with range where appropriate. Categorical variables are summarized as counts and percentages. Baseline characteristics between both groups were compared to assess comparability. Continuous variables were analyzed using Welch’s two-sample *t*-test to account for unequal variances and sample sizes, while categorical variables were compared using the Pearson chi-square test with Yates’ continuity correction or Fisher’s exact test where appropriate.

Radiographic alignment outcomes (HKA, MPTA, LDFA, aHKA, and JLO) were analyzed as continuous variables. Within-group changes from pre-operative to post-operative measurements were calculated, and between-group differences in change were assessed using Welch’s two-sample *t*-test. Rotational alignment, available only postoperatively, was compared directly between groups using Welch’s *t*-test. Longitudinal patient-reported outcome measures (PROMs), including Knee Society Score (KSS), Knee injury and Osteoarthritis Outcome Score (KOOS), and Visual Analogue Scale (VAS), were analyzed using linear mixed-effects models to account for repeated measurements within individuals over time. Fixed effects included treatment group, timepoint, and their interaction, while patient-level random intercepts were included to account for within-subject correlation. Where appropriate, post hoc pairwise comparisons between groups at each timepoint were performed with adjustment for multiple comparisons. The Forgotten Joint Score-12 (FJS-12), assessed at final follow-up, was compared between groups using the Wilcoxon rank-sum test due to non-normal distribution.

All statistical analyses were performed using R version 4.0.4 (R Foundation for Statistical Computing, Vienna, Austria) within the RStudio integrated development environment and a *p*-value <0.05 was considered statistically significant.

### Surgical technique

All surgeries were done utilizing a midline skin incision and medial parapatellar arthrotomy. For the conventional cohort, surgery was done in accordance with mechanical alignment (MA) principles, whereas Functional Alignment (FA) principles were adopted for the RATKA cohort [10, 11].

Cutting jig placement in conventional TKA surgeries was guided by standard intramedullary femoral and extramedullary tibial guides. For RATKA, intra-incisional fiducial pins were inserted into the femur and tibia after arthrotomy. This was followed by the registration of the hip centre and malleoli prior to the mapping of the distal femur and proximal tibia. Once anatomical accuracy was corroborated with the pre-operative CT scan, baseline native alignment and gap measurements were obtained before proceeding to the planning page. Bone cuts were adjusted in all three planes to attain satisfactory restoration of limb alignment and gaps within 1 mm between medial/lateral and flexion/extension gaps. The bone cuts were then completed by the surgeon with guidance from the robotic arm. Trials were used to confirm implant placement, gap balance and overall alignment.

All patients received a cemented Triathlon Cruciate Retaining Total Knee System (Stryker Orthopaedics, Mahwah, NJ) without patella resurfacing. No patients received drains. Full weight-bearing ambulation was commenced the morning after surgery. Outpatient physiotherapy was scheduled to commence one week after surgery to support rehabilitation.

## Results

### Baseline characteristics

A total of 45 patients were included (30 RATKA, 15 conventional). Baseline characteristics were comparable between both groups, except for height (*p* = 0.035), as detailed in Table 1.

**Table 1:** Baseline characteristics of patients undergoing conventional vs Robotic-assisted total knee arthroplasty.

Parameter	Conventional (*n* = 15)	Robotic (*n* = 30)	*p*-value
Gender			
Male	2 (13.33%)	13 (43.33%)	0.094
Female	13 (86.67%)	17 (56.67%)	
Laterality			
Right	10 (66.67%)	14 (46.67%)	0.342
Left	5 (33.33%)	16 (53.33%)	
Age (years)	67.40 ± 9.77	68.17 ± 5.73	0.782
Height (m)	1.54 ± 0.09	1.60 ± 0.09	0.035
Weight (kg)	69.87 ± 14.30	72.21 ± 12.10	0.592
BMI (kg/m^2^)	29.32 ± 4.47	28.14 ± 4.53	0.416
Surgery duration (minutes)	58.80 ± 10.83	62.97 ± 8.00	0.200

### Pain

**Figure 1:**
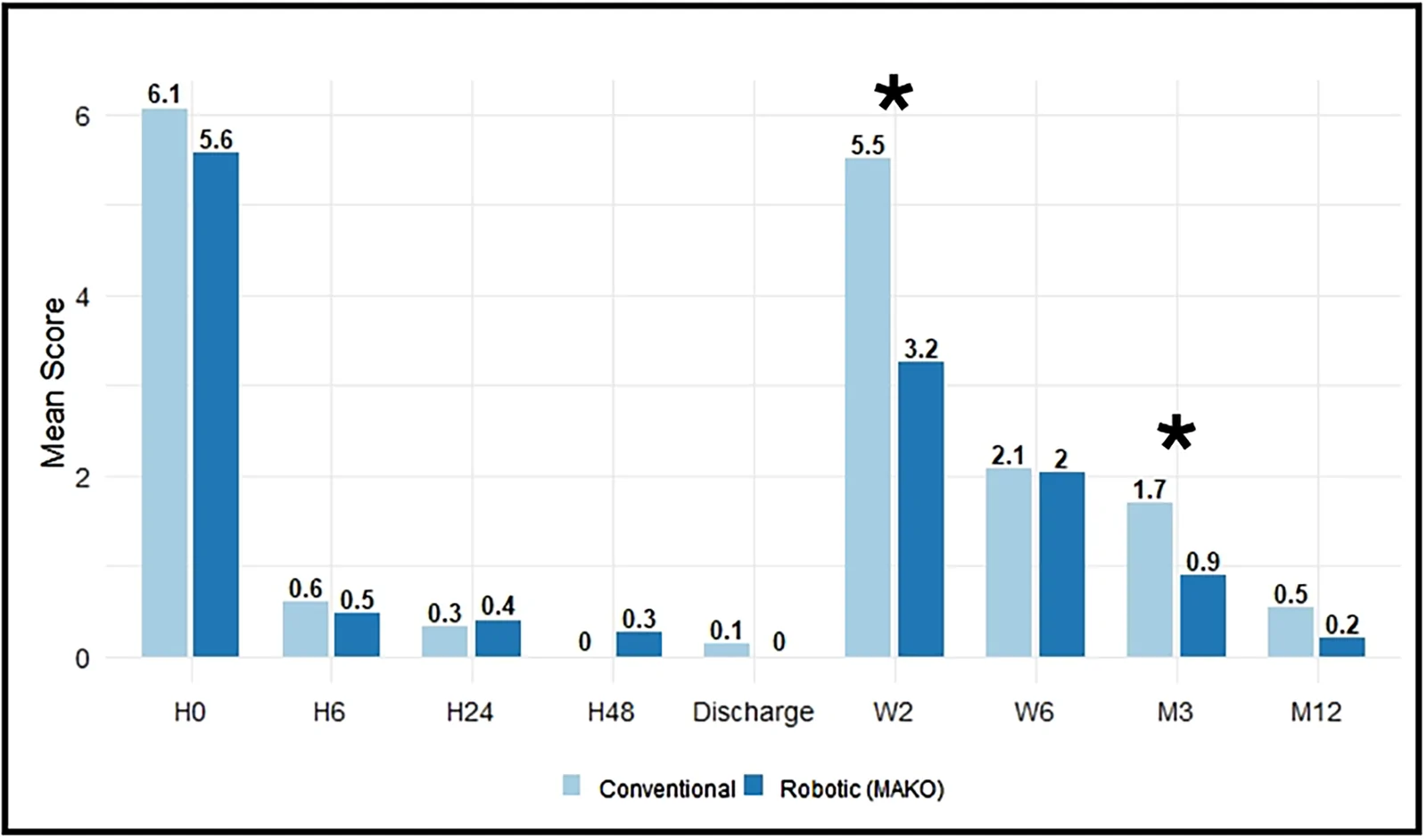
Pain score as measured using the Visual Analogue Scale (VAS). Mean scores are shown from immediate post-op (H0), 6 h post-op (H6), 24 h (H24), 48 h (H48), discharge, and subsequent outpatient clinic visits. * denotes statistical significance with *p* < 0.05.

RATKA demonstrated significantly lower VAS pain scores at 2 weeks (*p* < 0.001), with a consistent trend toward reduced pain at other timepoints. Figure 1 and Table 2 illustrate this better.

**Table 2:** Comparison of Knee Society Score (KSS) domains, Knee injury and Osteoarthritis Outcome Score (KOOS) domains, and Visual Analogue Scale (VAS) pain scores between Conventional and Robotic cohorts at each follow-up time point.

Domain	Timepoints	Conventional (Mean ± SD)	Robotic (Mean ± SD)	Mean difference (95% CI)	*p*-value
KSS					
Objective knee	Pre	33.0 ± 14.5	47.3 ± 17.1	14.3 (−24.2, −4.3)	**0.006**
	W2	59.4 ± 16.6	61.1 ± 14.8	1.7 (−12.1, 8.7)	0.740
	W6	62.4 ± 18.5	65.8 ± 14.2	3.4 (−14.7, 7.8)	0.533
	M3	61.6 ± 18.1	66.8 ± 14.5	5.2 (−16.4, 5.9)	0.341
	M12	61.7 ± 17.8	67.6 ± 14.2	5.8 (−16.7, 5.1)	0.280
Functional	Pre	26.5 ± 12.1	41.9 ± 15.2	15.3 (−23.8, −6.8)	**0.001**
	W2	25.9 ± 20.4	33.0 ± 13.4	7.1 (−19.7, 5.4)	0.228
	W6	42.5 ± 14.6	51.1 ± 14.3	8.6 (−18.1, 0.8)	0.071
	M3	57.8 ± 15.8	59.6 ± 14.2	1.8 (−12.4, 8.9)	0.734
	M12	75.5 ± 13.2	79.6 ± 11.7	4.1 (−12.4, 4.2)	0.319
Symptoms	Pre	6.8 ± 3.2	9.2 ± 5.8	2.4 (−5.1, 0.3)	0.075
	W2	10.4 ± 6.9	14.1 ± 5.4	3.8 (−8.1, 0.6)	0.086
	W6	17.8 ± 4.1	18.4 ± 3.4	0.6 (−3.2, 1.9)	0.609
	M3	19.3 ± 3.5	21.5 ± 3.8	2.2 (−4.7, 0.2)	0.076
	M12	22.7 ± 2.7	24.2 ± 1.5	1.5 (−3.1, 0.1)	0.060
Satisfaction	Pre	9.9 ± 4.1	12.1 ± 6.3	2.3 (−5.4, 0.9)	0.155
	W2	17.0 ± 8.3	21.8 ± 10.3	4.8 (−10.8, 1.2)	0.111
	W6	25.2 ± 8.4	29.7 ± 5.3	4.5 (−9.5, 0.4)	0.070
	M3	29.5 ± 5.5	32.3 ± 8.1	2.7 (−7.0, 1.6)	0.207
	M12	30.8 ± 5.6	34.9 ± 4.0	4.1 (−7.5, −0.7)	**0.021**
Expectations	Pre	13.1 ± 1.8	14.6 ± 1.2	1.6 (−2.7, −0.5)	**0.007**
	W2	8.4 ± 1.0	10.4 ± 2.7	2.0 (−3.2, −0.8)	**0.001**
	W6	10.1 ± 3.3	13.2 ± 2.6	3.2 (−5.2, −1.2)	**0.003**
	M3	10.9 ± 2.9	12.2 ± 2.9	1.3 (−3.3, 0.7)	0.182
	M12	8.4 ± 1.1	9.2 ± 0.8	0.8 (−1.5, −0.1)	**0.021**
KOOS					
Function, daily	Pre	39.9 ± 18.8	59.9 ± 18.1	20.1 (−32.1, −8.0)	**0.002**
	W2	39.3 ± 15.0	59.0 ± 15.6	19.7 (−29.9, −9.5)	**<0.001**
	W6	67.6 ± 13.6	75.9 ± 10.3	8.3 (−16.5, 0.0)	0.050
	M3	77.1 ± 12.5	85.5 ± 15.9	8.4 (−17.6, 0.8)	0.072
	M12	92.8 ± 5.7	92.3 ± 8.4	−0.5 (−3.8, 4.8)	0.818
Function, sports	Pre	10.0 ± 10.7	17.3 ± 11.3	7.3 (−14.4, −0.3)	**0.042**
	W2	10.7 ± 7.6	16.0 ± 20.8	5.3 (−14.1, 3.5)	0.230
	W6	21.3 ± 14.9	20.2 ± 15.2	−1.2 (−8.6, 10.9)	0.808
	M3	33.5 ± 19.2	37.2 ± 21.7	3.7 (−17.3, 9.9)	0.581
	M12	44.7 ± 15.3	68.3 ± 19.5	23.7 (−34.5, −12.9)	**<0.001**
Pain	Pre	42.0 ± 19.7	64.9 ± 14.2	23.0 (−34.8, −11.1)	**<0.001**
	W2	46.2 ± 17.8	62.7 ± 16.5	16.6 (−28.2, −4.9)	**0.007**
	W6	69.2 ± 16.3	78.3 ± 9.5	9.1 (−18.7, 0.4)	0.059
	M3	75.6 ± 11.5	84.8 ± 14.3	9.2 (−17.6, −0.8)	**0.034**
	M12	91.3 ± 6.2	93.3 ± 6.9	2.0 (−6.2, 2.1)	0.328
Symptoms	Pre	42.8 ± 15.1	65.1 ± 17.6	22.3 (−32.5, −12.0)	**<0.001**
	W2	50.8 ± 13.0	57.5 ± 14.8	6.7 (−15.7, 2.3)	0.141
	W6	62.9 ± 12.1	71.6 ± 10.3	8.8 (−16.3, −1.2)	**0.024**
	M3	71.2 ± 10.9	75.4 ± 17.2	4.2 (−13.1, 4.7)	0.346
	M12	87.5 ± 7.8	90.0 ± 8.5	2.5 (−7.7, 2.7)	0.327
QOL	Pre	31.7 ± 16.8	44.2 ± 19.9	12.5 (−24.0, −1.0)	**0.035**
	W2	36.6 ± 16.5	50.9 ± 17.2	14.3 (−25.4, −3.1)	**0.014**
	W6	52.1 ± 13.9	64.6 ± 13.4	12.5 (−21.4, −3.6)	**0.008**
	M3	60.7 ± 11.9	70.0 ± 21.3	9.3 (−19.7, 1.0)	0.075
	M12	88.8 ± 9.2	85.2 ± 9.9	−3.6 (−2.5, 9.7)	0.243
Pain (Visual Analogue Scale, VAS)					
H0		6.1 ± 2.0	5.6 ± 2.6	− 0.5 (−0.9, 1.9)	0.485
H6		0.6 ± 0.8	0.5 ± 1.0	−0.1 (−0.4, 0.7)	0.640
H24		0.3 ± 0.8	0.4 ± 0.9	0.1 (−0.6, 0.5)	0.801
H48		0.0 ± 0.0	0.3 ± 0.7	0.3 (−0.5, 0.0)	**0.043**
Discharge		0.1 ± 0.4	0.0 ± 0.0	−0.1 (−0.1, 0.3)	0.164
W2		5.5 ± 1.7	3.2 ± 2.0	−2.2 (1.0, 3.5)	**<0.001**
W6		2.1 ± 1.7	2.0 ± 1.7	−0.0 (−1.1, 1.1)	0.955
M3		1.7 ± 1.2	0.9 ± 1.3	−0.8 (−0.1, 1.6)	**0.064**
M12		0.5 ± 0.8	0.2 ± 0.5	−0.3 (−0.2, 0.8)	0.168

*Pre-operative*; *H* Hour; *W* Week; *M* Month; *QOL* Quality of Life.

### Patient reported outcome measures

**Figure 2:**
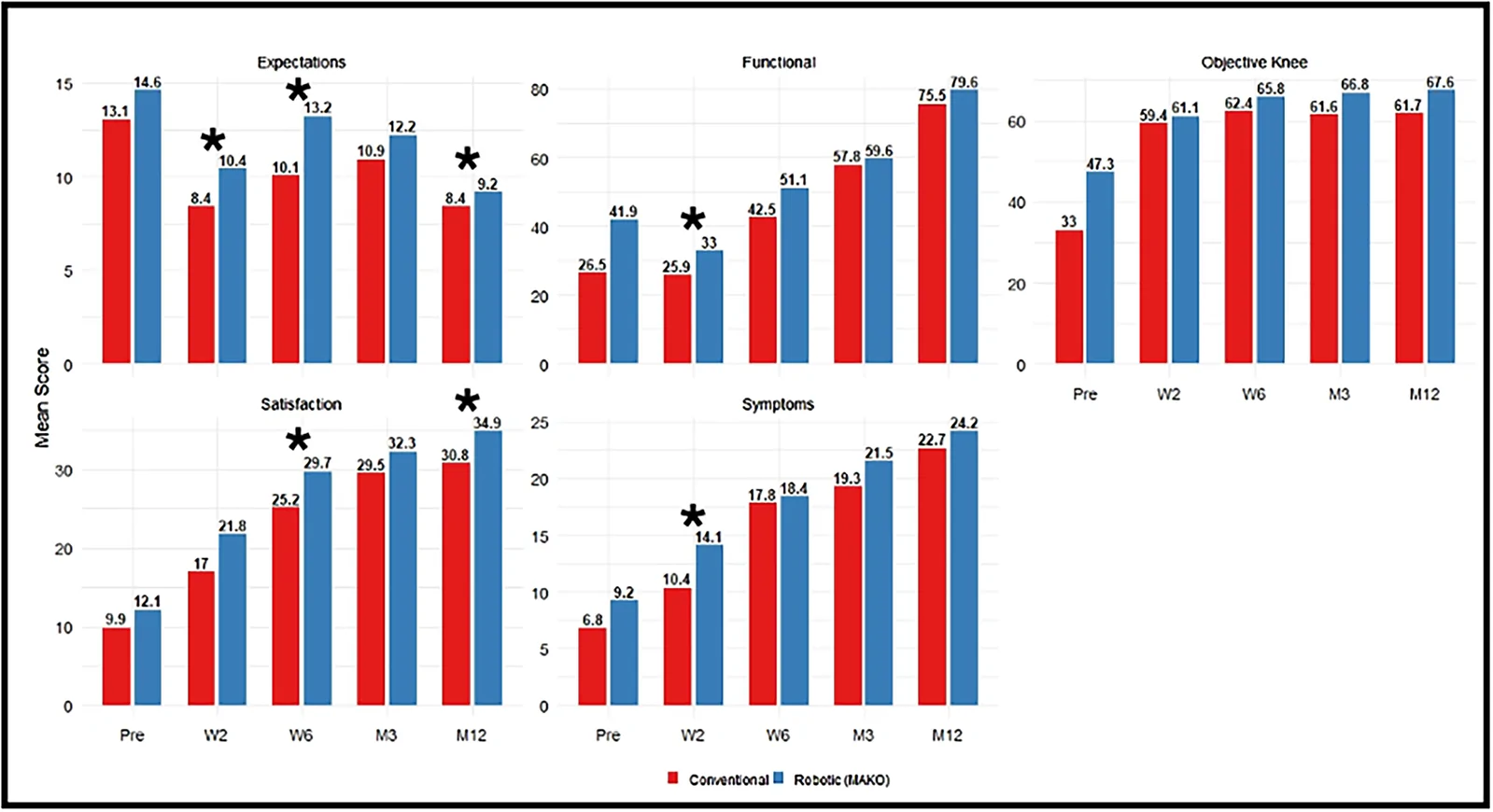
Longitudinal Knee Society Score (KSS) broken down into subcategories. Serial results are shown from preoperative baseline, 2 weeks post-op (W2), 6 weeks (W6), 3 months (M3), and 1 year (M12). * denotes statistical significance with *p* < 0.05.

**Figure 3:**
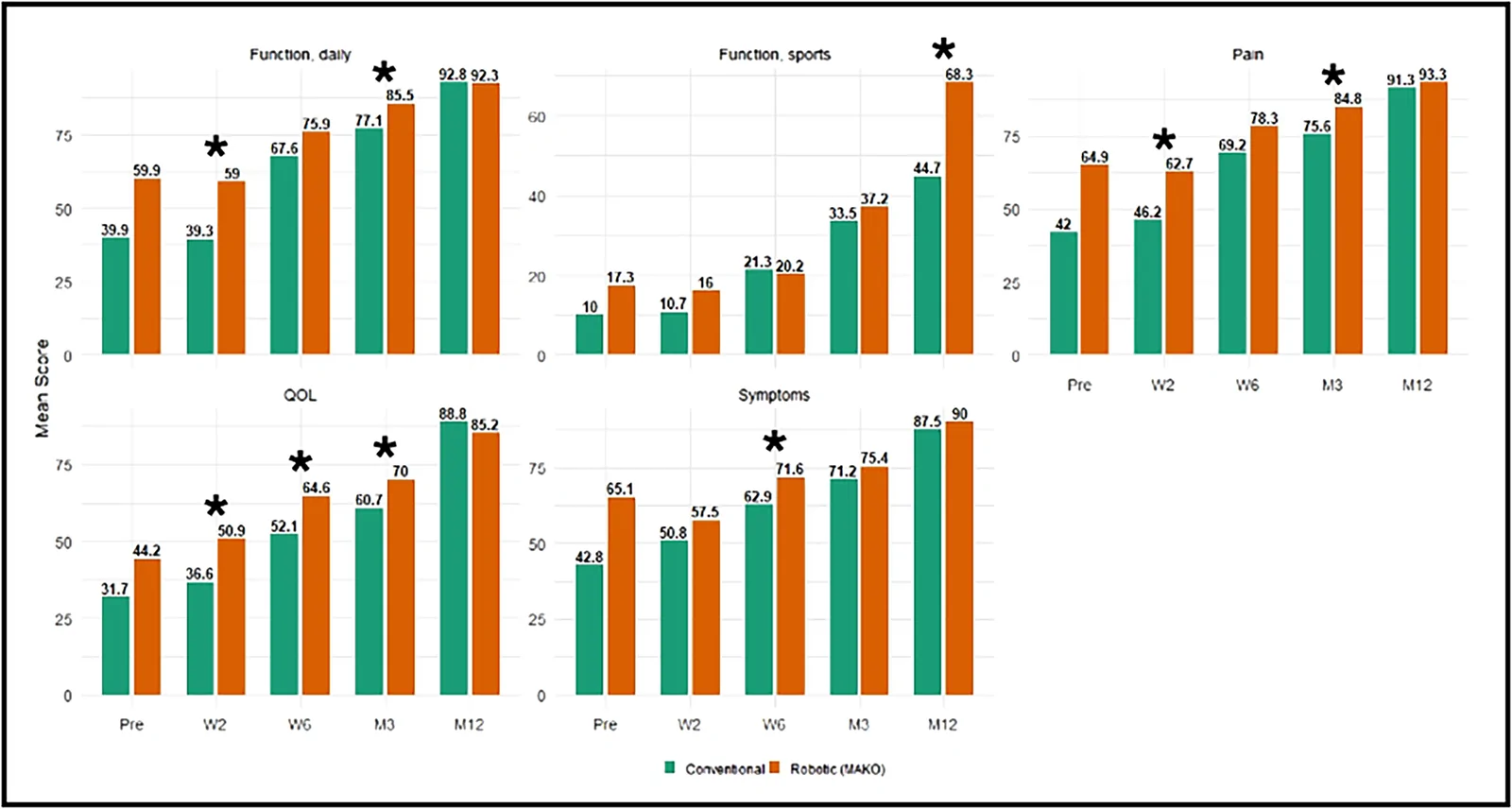
Longitudinal Knee Injury and Osteoarthritis Outcome Score (KOOS) broken down into subcategories. Serial results are shown from preoperative baseline, 2 weeks post-op (W2), 6 weeks (W6), 3 months (M3), and 1 year (M12). * denotes statistical significance with *p* < 0.05.

PROMs showed improvements in both groups. RATKA demonstrated statistically significant advantages in selected KSS (Figure 2, Table 2) and KOOS (Figure 3, Table 2) subdomains at specific timepoints.

**Figure 4:**
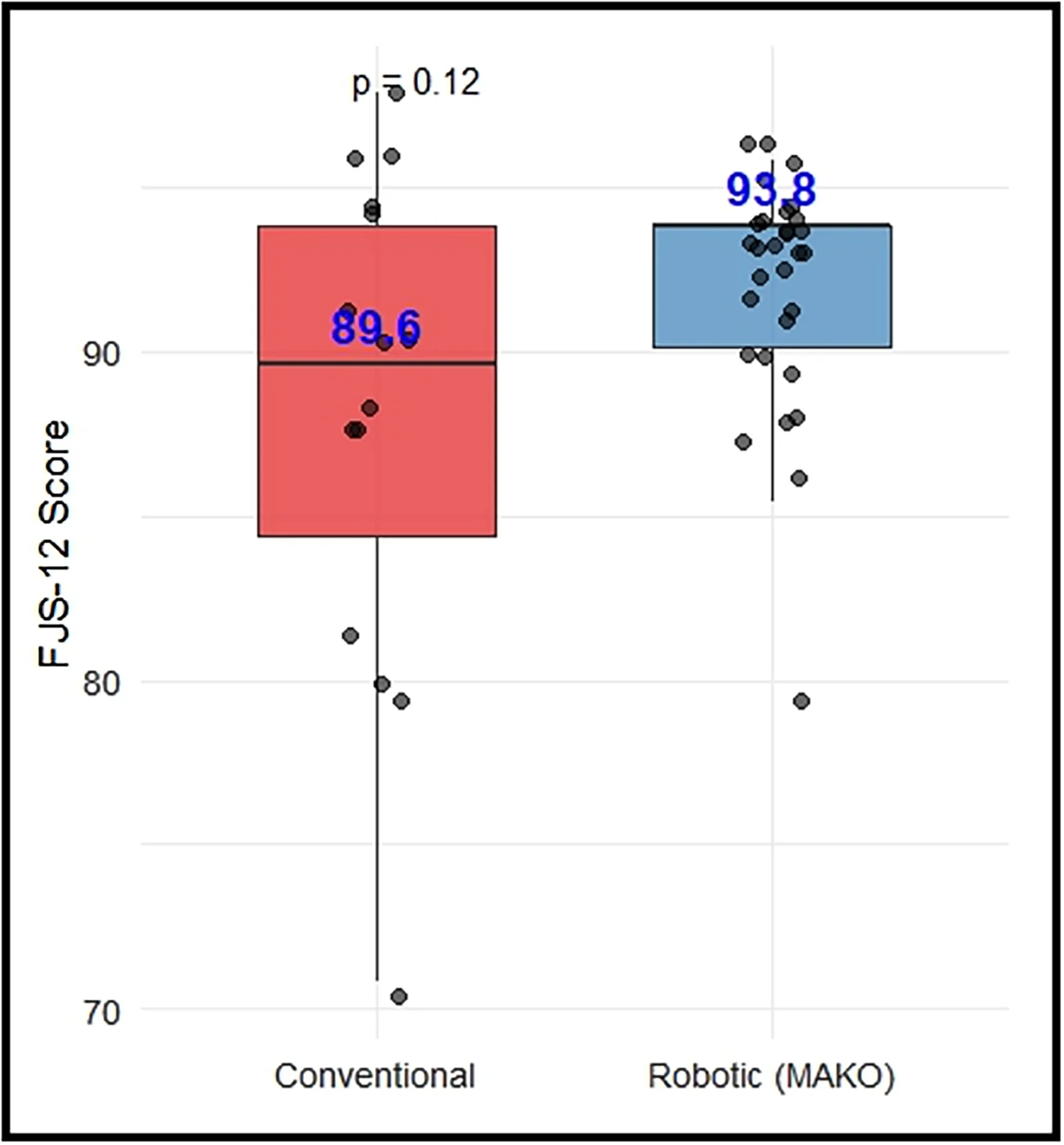
Forgotten Joint Score-12 comparison between both cohorts. Boxplots represent the median and interquartile range, with individual patient data points overlaid. Blue numbers denote median values. Between-group comparison was performed using the Wilcoxon rank-sum test.

The median FJS score of the Robotic cohort was higher than that of the Conventional cohort, with a tighter interquartile range as shown in Figure 4 below. However, this did not achieve statistical significance, *p* = 0.12.

### Alignment

Radiographic alignment was comparable between groups, except for femoral component rotation, which demonstrated greater external rotation in the conventional group (0.94 ± 0.42 vs 0.59 ± 0.23, *p* = 0.009). Table 3 details the comparison comprehensively.

**Table 3:** Radiographic alignment outcomes following total knee arthroplasty, comparing Conventional and Robotic techniques.

	Preoperative (Mean ± SD)	Postoperative (Mean ± SD)	Δ Change (Pre – Post)	Between-Group Difference (Conventional – Robotic)	*p*-value
*HKA*					
Conventional	−10.46 ± 4.93	−2.82 ± 1.89	7.64 ± 4.40	−0.35 ± 9.28	0.800
Robotic	−9.80 ± 5.03	−1.80 ± 1.66	7.99 ± 4.31		
*MPTA*					
Conventional	84.77 ± 3.62	89.09 ± 2.09	4.32 ± 3.55	0.60 ± 7.15	0.581
Robotic	84.41 ± 2.92	89.32 ± 1.61	4.92 ± 2.99		
*LDFA*					
Conventional	89.11 ± 3.24	91.41 ± 2.43	2.31 ± 3.12	−0.86 ± 6.03	0.351
Robotic	88.75 ± 2.45	90.20 ± 1.41	1.45 ± 2.18		
*aHKA*					
Conventional	5.71 ± 3.90	3.19 ± 1.99	−2.52 ± 3.03	−1.01 ± 6.69	0.321
Robotic	5.16 ± 3.68	1.63 ± 1.68	−3.53 ± 3.39		
*JLO*					
Conventional	173.88 ± 4.17	180.51 ± 3.38	6.63 ± 5.55	−0.26 ± 10.49	0.870
Robotic	173.15 ± 2.75	179.52 ± 2.10	6.37 ± 3.44		
*Rotational*					
Conventional		0.94 ± 0.42		0.35 ± 0.12	0.009
Robotic		0.59 ± 0.23			

*HKA* hip-knee-ankle axis; *MPTA* medial proximal tibial angle; *LDFA* lateral distal femoral angle; *aHKA* arithmetic hip-knee-ankle angle; *JLO* joint line obliquity.

The proportion of alignment outliers was significantly lower in the RATKA group (10.0% vs 40.0%, *p* = 0.042), corresponding to a 75% relative risk reduction.

### Complications

There were no cases of wound dehiscence, superficial skin infection, prosthetic joint infection, foot drop, neurovascular injury, deep vein thrombosis, pulmonary embolism, or prolonged knee swelling that necessitated drainage for both cohorts of this study. Additionally, there were also no complications related to tracker pin arrays such as pin site fractures, broken or retained pins, nor wound issues. 90-day and 1-year readmission rates were zero for this study.

## Discussion

In this prospective cohort study, RATKA was associated with reduced early postoperative pain, modest improvements in PROMs, and a significantly lower rate of alignment outliers compared to conventional TKA, without an increase in complications. These findings support the role of robotic assistance in enhancing early recovery and improving the precision of implant positioning.

The reduction in early postoperative pain observed in the RATKA cohort is likely multifactorial. The adoption of a functional alignment philosophy [10, 11], facilitated by robotic technology, enables precise bone resections and objective gap balancing with reduced reliance on soft tissue releases. This minimizes peri-articular soft tissue trauma and contributes to the reduction in postoperative pain. Our findings corroborate the findings of Kayani, who in a prospective cohort study compared 40 patients undergoing RATKA with another 40 patients undergoing conventional TKA and found that the RATKA arm had lower pain scores and opioid consumption [12]. Similarly, Bhimani et al. performed a retrospective analysis on 268 patients from their institution’s joint registry and found that the RATKA patients had reduced pain scores both at rest and during activity at 2 weeks and 6 weeks post-surgery [13]. Importantly, improved early pain control may facilitate rehabilitation, enhance patient engagement, and potentially translate into improved overall satisfaction.

PROMs demonstrated a general trend favoring RATKA across multiple KSS and KOOS subdomains, with statistically significant differences observed at selected timepoints. While these findings align with prior studies reporting improved functional outcomes with robotic assistance, the magnitude of differences should be interpreted cautiously. Stoltz conducted a retrospective study comparing 393 RATKAs vs 312 manual TKAs with a minimum of 2-years of follow-up and reported a statistically significant difference in KSS Function and Knee scores, favouring the RATKA patients [14]. In a same-patient retrospective comparative analysis on 36 patients who initially underwent conventional TKA on one knee before undertaking a RATKA on the contralateral knee, Ali reported that the mean KOOS score was higher for the RATKA knees, which also had expedited improvements in pain, stiffness and knee flexion [15]. Both cohorts in our study achieved the minimum clinically important difference (MCID) [16, 17] by three months postoperatively, suggesting that while RATKA may accelerate early recovery, longer-term functional outcomes may ultimately converge.

Radiographic analysis demonstrated comparable restoration of coronal alignment between groups; however, RATKA resulted in a significantly lower proportion of alignment outliers. This agrees with the findings of Lee et al., who reported a reduced outlier rate for the RATKA arm of his study compared to the conventional and navigated TKA groups (9.5% vs 25.7%, 14.1%, *p* < 0.001) [18]. In Nam’s retrospective propensity score-matched analysis comparing 110 patients who undertook a MAKO RATKA vs 110 conventional TKAs, the outlier rate for postoperative HKA was 18.2% and 35.7%, respectively, and this was statistically significant, *p* < 0.05 [19]. This finding is clinically relevant, as deviations from planned alignment have been associated with inferior functional outcomes and reduced implant longevity. The improved consistency observed with RATKA likely reflects enhanced preoperative planning and intra-operative execution enabled by real-time feedback and haptic guidance.

A notable finding was the increased external rotation of the femoral component in the conventional cohort. This likely reflects reliance on traditional anatomical landmarks and surgical heuristics to achieve balanced flexion gaps and improve patellar tracking. In contrast, RATKA allows objective intra-operative gap assessment and kinematics, reducing the need for such adjustments. The ability to individualize femoral component rotation based on quantitative data represents a non-insignificant advantage of robotic systems.

No early complications were observed in either cohort, including those specific to robotic systems such as tracker pin-related issues. While this supports the safety of RATKA, the relatively small sample size is underpowered to detect infrequent adverse events.

This study has several limitations. First, the sample size is modest and may be underpowered to detect smaller but clinically relevant differences, particularly in PROMs. Next, although gender distribution did not attain statistical significance, there exists a difference, with females accounting for 86.67% of the conventional arm whereas this was only 56.67% in the RATKA cohort. There was also a statistically significant difference in height between both arms. These could be potential confounders, and the results of this study should be interpreted with these in mind. The non-randomized design introduces potential selection bias, especially in a private healthcare setting where patient preference influences treatment allocation. The unfortunate inability to randomize patients may introduce elements of expectation and socioeconomic bias. Next, the follow-up period is limited to one year, precluding assessment of long-term outcomes and implant survivorship. Additionally, the clinical assessor was not blinded; however, in our patients, the robotic tracker pins are placed intra-incisionally, and the surgical scars are identical for all patients, thus reducing assessment/observer bias. Another limitation would be the adoption of differing alignment philosophies for both cohorts (FA for the RATKA arm and MA for the conventional arm), which prevents an isolated comparison of the independent effects brought about by robotic assistance. However, we opine that adopting MA when utilizing RATKA does not fully harness its benefit and ergo be unfair to the patients undergoing RATKA. Next, the study was conducted at a single institution, which may limit generalizability. Nevertheless, strengths of this study include its prospective design, standardized surgical technique performed by a single experienced surgeon, and comprehensive radiographic evaluation including computed tomography-based assessment of rotational alignment.

## Conclusion

Robotic-assisted total knee arthroplasty is associated with reduced early postoperative pain, small but significant improvements in patient-reported outcomes, and improved alignment consistency with fewer outliers, without increased complications. Further large-scale studies with long-term follow-up are required to determine the clinical significance of these findings.

## Data Availability

The data that support the findings of this study are not publicly available due to patient privacy and confidentiality but are available from the corresponding author on reasonable request.
